# Effect of larval density and substrate quality on the wing geometry of *Stomoxys calcitrans* L. (Diptera: Muscidae)

**DOI:** 10.1186/s13071-019-3483-y

**Published:** 2019-05-10

**Authors:** Steve B. S. Baleba, Daniel Masiga, Baldwyn Torto, Christopher W. Weldon, Merid N. Getahun

**Affiliations:** 1 International Centre of Insect Physiology and Ecology (icipe), P.O. Box 30772-00100, Nairobi, Kenya; 2 Department of Zoology and Entomology, University of Pretoria, Private Bag X20, Hatfield, 0028 South Africa

**Keywords:** *Stomoxys calcitrans*, Wing morphology, Larval density, Developmental substrate, Geometric morphometrics, Phenotypic plasticity

## Abstract

**Background:**

In insects, oviposition decisions may lead to egg deposition in substrates with different larval density and nutritional levels. Individuals developing in such substrates may present plasticity in their phenotype. Here, we investigated the effect of two factors related to oviposition decisions, namely larval density and substrate quality, on the wing size and wing shape of the stable fly, *Stomoxys calcitrans* L. (Diptera: Muscidae).

**Methods:**

We reared *S. calcitrans* larvae at different densities (5, 15 and 25) and on different substrates (camel, cow, donkey and sheep dung). For each fly that emerged, we recorded body weight, and detached, slide-mounted and photographed the right wing. Next, we collected 15 landmarks on each photographed wing, and applied geometric morphometric analysis to assess variation in wing size and wing shape of *S. calcitrans* across the different larval densities and substrate types.

**Results:**

We observed that wing size and wing shape of *S. calcitrans* were affected by larval density and the nature of the developmental substrate. Flies reared in a group of 5 had larger wing centroid size, wing length, wing width, wing area and wing loading compared with those reared in a group of 25. Also, flies developed in donkey and sheep dung had larger wing centroid size, wing length, wing width, wing area and wing loading in comparison with those grown in camel and cow dung. Canonical variate analysis followed by discriminant analysis revealed significant wing shape variation in *S. calcitrans* across the different densities and substrates. Wing size had a significant but weak positive effect on wing shape.

**Conclusions:**

This study demonstrates the high sensitivity of *S. calcitrans* wings to variation in larval density and developmental substrate, and that use of landmark-based geometric morphometric analysis could improve our understanding of how flies of veterinary importance respond to environmental variability.

## Background

In holometabolous insects, individual fitness mostly relies on oviposition decisions by gravid females. When, where and how mothers deposit their eggs can affect the performance and phenotype of their progeny [1]. Therefore, it is important for females to oviposit on a substrate that provides the best conditions for the next generation. However, a female may fail to make the seemingly optimal choice to oviposit on an appropriate substrate that could enhance offspring fitness. For instance, Heard [2] found that in the pitcher plant mosquito, *Wyeomyia smithii* Coquillett, although larval fitness is better in pitchers with fewer conspecific and more midge larvae, gravid females did not deposit more eggs in such pitchers. Instead, they laid more eggs in pitchers containing either midges or conspecific larvae. Wong et al. [3] found that larval survival and development of *Aedes aegypti* L. was poor in containers where gravid females laid more eggs. This imperfection in oviposition decisions generally leads to phenotypic variation [4, 5], in which individuals react to the inputs of their breeding substrate with a change in their form, state, movement, or rate of activity [6]. These inputs include environmental factors such as the dietary value of the substrate and the number of individuals sharing the same substrate (density) [7]. In evolutionary ecology, understanding how these factors influence organism phenotype is a fundamental concern because such flexibility can affect fitness, generate novelty, facilitate evolution, and structure ecological communities [8].

Insect wings are good indicators of population responses to changes that occur in their environment [9]. Lin et al. [10] demonstrated that variation in food nutrient content and density are key ecological factors related to the expression of condition-dependent, adaptive phenotypes such as wing polyphenisms. They found that in the brown planthopper, *Nilaparvata lugens* Stål, a serious rice pest, an increase of long-winged *N. lugens* in a population is related to higher glucose levels in host rice plants. Conversely, the appearance of the short-winged form of *N. lugens* is linked to a reduction in host glucose level. Dipteran species are particularly well suited for studying phenotypic changes induced by the environment because their wings are highly plastic and wing landmarks are homologous across various species [11]. Variation in food quality and population density are key factors associated with fly wing polyphenisms. In *Drosophila buzzatii* Patterson & Wheeler and *Drosophila koepferae* Soto et al. [12] detected significant differences in wing size and shape between flies that were reared on different cactus hosts. In *Ae. aegypti*, males and females have longer wings when developed in conditions of low larval density [13].

Changes in wing morphology are known to affect insect flight aerodynamics. Long and slender wings are optimal for long-duration flight, while short and broad wings are optimal for slow and agile flight [14]. Also, broad wing bases allow a wider range of speed and a narrow wing tip allows less costly, extensive flight [15]. As a consequence, wing morphology is closely related to several insect behavioural activities including food searching, location of breeding sites and sexual partners, and avoidance of natural enemies. In some mosquito species such as *Anopheles gambiae* Giles where wing size is positively correlated with body size, an increase in wing size augments the frequency of blood meals [16]. This then leads to an increase in the likelihood of pathogen dissemination [16]. In *Aedes albopictus* Skuse, there is a positive correlation between wing length, larval diet quality, and the number of eggs laid [17]. It has been demonstrated that males of the olive fruit fly, *Bactrocera oleae* Rossi, with large wings (characterised by a high vibration frequency) achieve higher mating success than males with smaller wings [18].

Fly wing vein networks are excellent models for statistical analysis of size and shape variation [19]. In recent years, landmark-based geometric morphometric analysis has been increasingly used to analyse insect wings to address intraspecific variation [20, 21], interspecific variation [22, 23], sexual dimorphism [24, 25], parasite detection [26, 27], laboratory strain separation [28] and phenotypic plasticity [12, 29, 30]. Geometric morphometric techniques are potent tools to assess the correlation between the size and shape of organisms and environmental variables. The approach uses coordinates of identified morphological “landmarks” to study the form of biological structures in two or three dimensions. It involves several statistical techniques that preserve shape information and detect even subtle morphological variations [31]. Moreover, geometric morphometric techniques are cheap, simple and fast [32]. Using geometric morphometric analysis, this study examined the changes that occur in the wings of the stable fly, *Stomoxys calictrans* L. (Diptera: Muscidae) reared on different substrates and over a range of larval densities.

*Stomoxys calcitrans* is a cosmopolitan haematophagous fly that mechanically transmits viruses (e.g. West Nile fever virus, Rift Valley fever virus), bacteria (e.g. *Bacillus anthracis*, *Pasteurella multocida*), protozoans (e.g. *Trypanosoma evansi*, *Besnoitia besnoiti*), and helminths (e.g. *Habronema microstoma*, *Dirofilaria repens*) to their hosts, which include cattle, camels, horses, dogs, and humans [33–35]. During outbreaks, *S. calcitrans* can reduce weight gain in cattle by up to 19%, and lead to a 40–60% reduction in milk yields [36, 37]. In the USA, Taylor et al. [38] estimated economic losses attributable to *S. calcitrans* infestation at around $2.211 billion per year. Gravid female *S. calcitrans* oviposit on vertebrate herbivore dung including that of camel, cow, donkey and sheep, with the latter two the most preferred [39]. It has already been demonstrated that the fitness of *S. calcitrans* immature stages (hatchability, developmental time, emergence time, larval and pupal weight) varies across these substrates due to differences in their physicochemical composition [39]. However, the way in which preferred and non-preferred substrates affect *S. calcitrans* wing size and wing shape remains unclear. Furthermore, not only the substrate nutrient quality but also larval density should be assessed. We hypothesised that larval density and vertebrate herbivore dung type on which *S. calcitrans* develop would affect wing size and shape.

## Methods

### Biological material

*Stomoxys calcitrans* flies were obtained from a single culture that had been established for approximately 8 months at the Duduville campus of the International Centre for Insect Physiology and Ecology (icipe) in Nairobi (1°13′12″S, 36°52′48″ E; *c.*1600 m above sea level). By sourcing experimental flies from a laboratory culture, we minimised potential variation between populations. Adults reared from rabbit faeces were kept in cages (75 × 60 × 45 cm) under conditions of 25 ± 5 °C and 65 ± 5% relative humidity with a photoperiod of 12L:12D. Flies were fed twice per day (8:00 and 16:00 h) on defibrinated bovine blood poured on moistened cotton.

### Density experiment

Gravid female *S. calcitrans* from the established colony were allowed to oviposit on donkey dung placed in plastic containers (21.5 × 14.5 × 7.4 cm). Baleba et al. [39] demonstrated that this dung is best for *S. calcitrans* development. To assess the effect of density of *S. calcitrans* on wing size and shape, we reared *S. calcitrans* larvae at varying densities by gently transferring (using soft forceps) 5, 15 and 25 first-instar larvae to plastic cups (200 ml) filled with 25 g of donkey dung. We replicated this process several times to obtain 30 emerged females and 30 emerged males. After emergence, each individual was weighed, killed in 70% ethanol, and its right wing was gently removed from the thorax using a fine clamp. The removed wings were slide-mounted (dorsally placed between two microscope slides) to avoid deformation and to enhance accuracy during photography and landmark collections [40]. We photographed the wings at 16× magnification with a Leica DFC320 digital camera coupled to a Leica S6 microscope.

### Substrate quality experiment

To test the effect of substrate on *S. calcitrans* wing size and shape, 10 first-instar larvae were transferred to and permitted to develop on 25 g of camel, cow, donkey or sheep dung. It has been shown that these substrates differ in their physico-chemical composition and affect development and adult body weight [39]. We replicated the process several times to obtain 30 emerged females and 30 emerged males. After emergence, each individual of *S. calcitrans* was weighed, killed in 70% ethanol, the right wing was removed and slide-mounted, and wings were photographed as described above.

### Wing geometric morphometric analysis

**Fig. 1:**
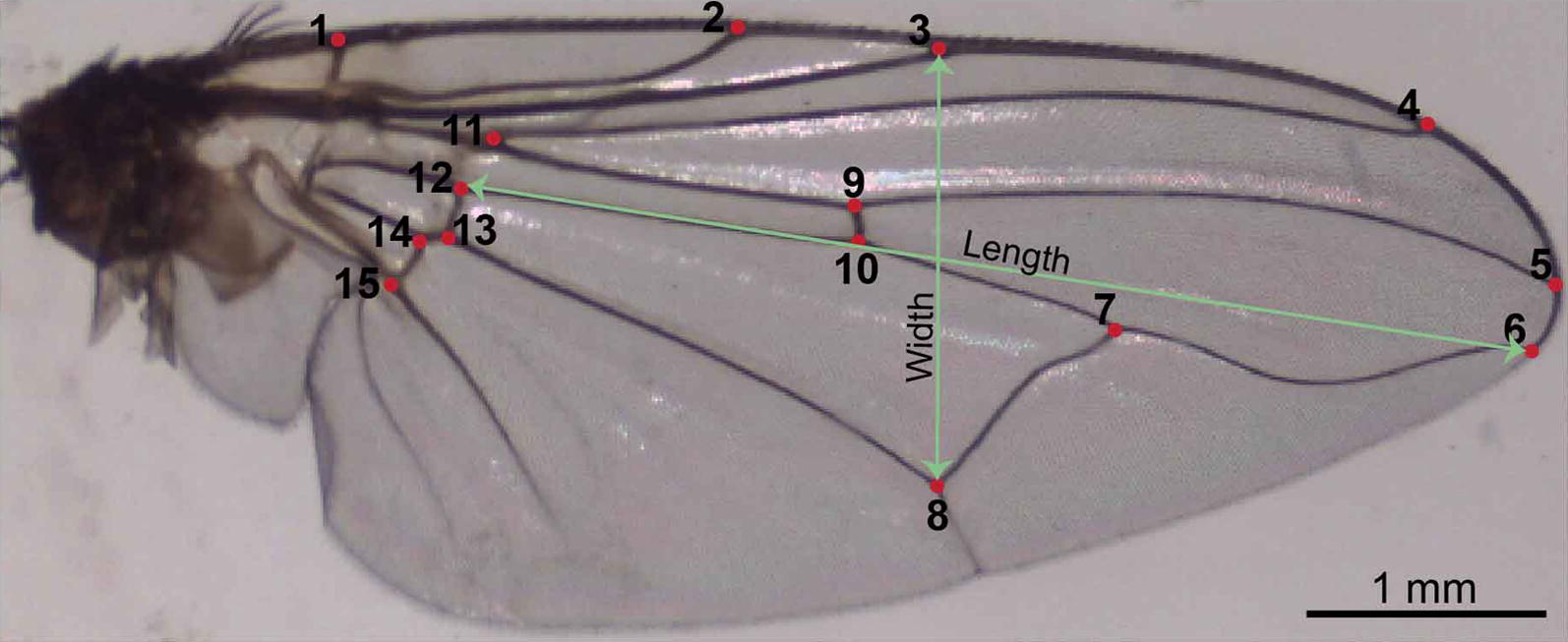
Dorsal view of the right wing of *S. calcitrans*. Numbers indicate the location of 15 selected landmarks (described in Table 1)

To collect wing landmark coordinates, we opened the digital photographs in ImageJ software [41] and generated Cartesian coordinates for 15 wing landmarks (Fig. 1). To quantify measurement error relative to the landmark digitalisation, we collected landmarks for the wings of all 30 individuals reared from donkey dung three times. After executing the generalised Procrustes analysis to extract shape information from the data and eliminate differences in orientation, position and isometric size, we ran analysis of variance (ANOVA) and multivariate analysis of variance (MANOVA) tests to determine if wing size and the wing shape of *S. calcitrans* varied across the three landmark collections [42] (Table 1).

**Table 1:** Description of the 15 anatomical landmarks used to characterise *S. calcitrans* wing geometry. Numbers relate to landmarks shown in Fig. 1

Anatomic position of landmark	Description
1	Costal vein intersection with humeral vein
2	Costal vein intersection with subcosta vein
3	Costal vein intersection with radial vein 1
4	Costal vein intersection with radial vein 2+3
5	Costal vein intersection with radial vein 4+5
6	Costal vein intersection Medial vein
7	Basal median cubital vein intersection medial vein
8	Basal median cubital vein and anterior cubital 2
9	Radial vein 4+5 vein intersection with radial medial
10	Medial vein intersection with radial medial vein
11	Radial 2+3 vein intersection with radial 4+5 vein
12	Medial vein intersection with distal median cubital
13	Anterior cubital vein 1 intersection with distal median cubital
14	Anterior cubital vein 1 intersection with anterior cubital vein 2
15	Anal vein 1 intersection with anterior cubital vein 2

Four parameters were derived to describe wing size of *S. calcitrans*: (i) centroid size; (ii) wing length (distance between the 6th and 12th landmark); (iii) wing width (distance between the 3rd and 8th landmark) (Fig. 1); and (iv) wing area. The centroid size, also called the “configuration barycentre” is a global size (or multidimensional measurement) calculated as the square root of the sum of squared Euclidean distances between each landmark and the wing centroid. We computed centroid size using PAST software V.3.09 [43]. Also, based on the adult weight parameter, we calculated wing loading (in kg/m^2^) using the formula: wl = mass/wing area [44]. To assess the effect of larval density (5, 15 and 25) and substrate type (camel, cow, donkey and sheep dung) on the parameters described above, we ran analyses of variance (ANOVA) followed by *post-hoc* Student-Newman-Keuls (SNK) tests after checking the wing size parameters for normality using the Shapiro-Wilk test (P > 0.05). To identify correlations between centroid size, wing length, wing width, wing area, adult weight and wing loading, we performed separate principal components analysis (PCA) for larval density and substrate type. We used R version 3.5.1 software [45] to compute all statistical analyses.

To assess wing shape variation across the different densities and substrates, we imported the raw landmark Cartesian coordinates into MorphoJ software [46]. This software was first used to perform a generalised Procrustes analysis to extract shape information from the data and eliminate differences in orientation, position and isometric size. Afterwards, we ran separate multivariate analyses of variance (MANOVA) to compare wing shapes across the different larval densities (5, 15 and 25) and substrates (camel, cow, donkey and sheep dung). Using PAST software, we performed thin plate spline analysis to visualise wing shape deformations. We used canonical variate analysis combined with discriminant analysis to analyse the relative similarities and dissimilarities of the different wing groups. To determine the significance of pairwise differences in mean shapes, we performed permutation tests (10,000 rounds) with Mahalanobis distances and Procrustes distances. To assess the effect of wing size on wing shape (allometry), we fit a linear regression between the Procrustes coordinates and the centroid size, using a permutation test with 10,000 randomisations. For all of these analyses, we excluded the effect of sex because preliminary analyses indicated that wing shape of females and males did not differ.

## Results

### Measurement error test

The landmarks measured repeatedly on the same individual wing (3 times) were not significantly different for both size (ANOVA, *F*_(2,84)_ = 0.03; *P* = 0.97) and shape (MANOVA, *F*_(48,2016)_ = 0.6; Pillai’s trace = 0.16; *P* = 0.99). Therefore, we ruled out error due to landmark digitalisation, and we considered that any differences found in the morphology of *S. calcitrans* wings resulted from the two factors manipulated in our study (larval density and substrate type).

### Effect of larval density on the wing geometry of *S. calcitrans*

#### Wing size parameters

**Fig. 2:**
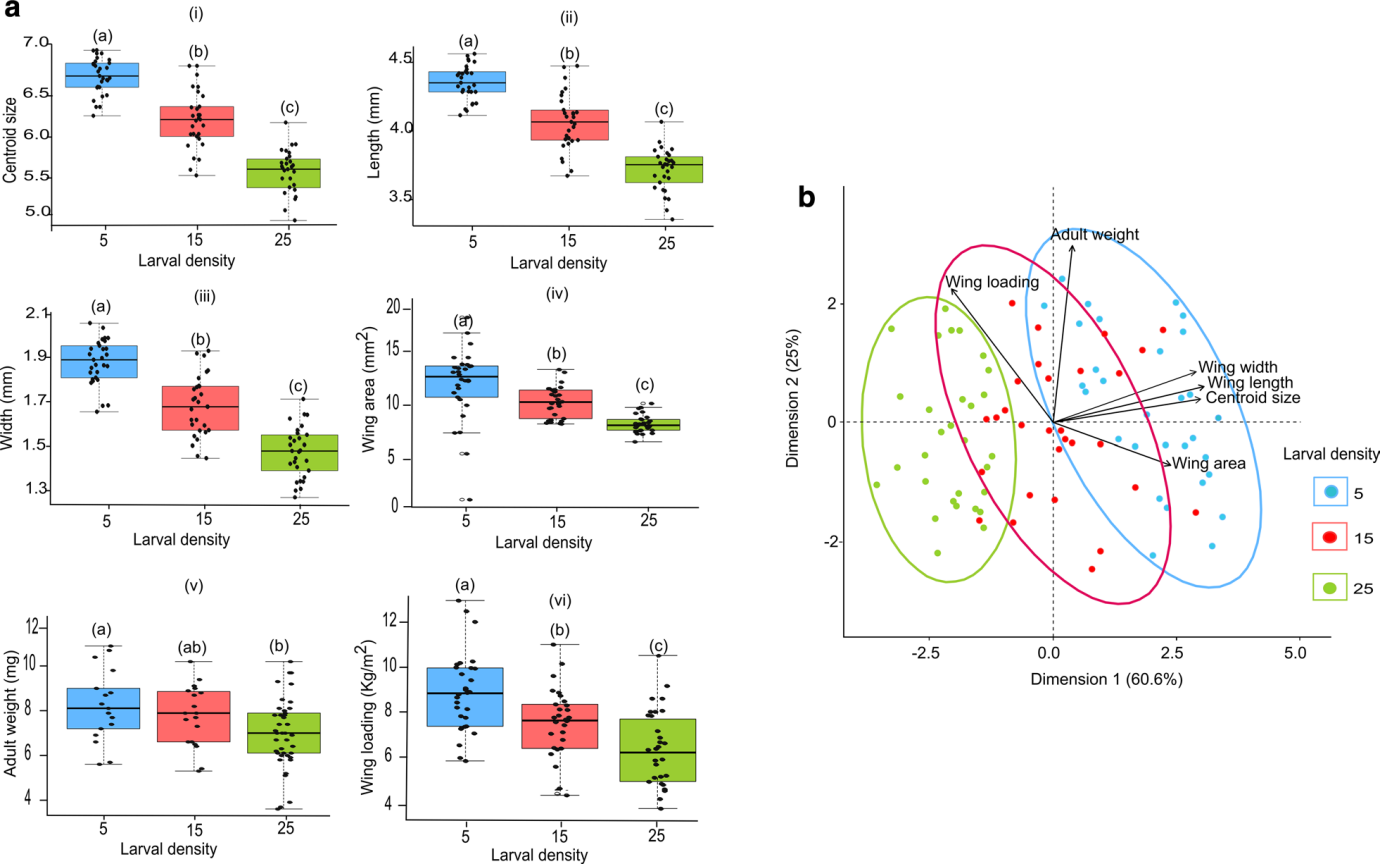
Wing size and adult weight of *S. calcitrans* is significantly affected by larval density. **a** Boxplots depicting variation in wing centroid size (i), wing length (ii), wing width (iii), wing area (iv), adult weight (v), and wing loading (vi) across the different larval densities. Boxplot whiskers indicate ± 1.5 interquartile range limits. Boxplots with different letters show significant differences as grouped by ANOVA tests followed by SNK *post-hoc* tests (*P *< 0.05*, n* = 30). **b** Principal components biplot showing the similarities (or dissimilarities) existing among flies reared at different densities (5, 15 and 25)

Larval density significantly affected wing centroid size (*F*_(2,84)_ = 104.1, *P *< 0.0001), wing length (*F*_(2,84)_ = 97.91, *P *< 0.0001), wing width (*F*_(2,84)_ = 85.63, *P *< 0.0001), wing area (*F*_(2,84)_ = 22.67, *P *< 0.0001), adult weight (*F*_(2,84)_ = 4.51, *P* = 0.014) and wing loading (*F*_(2,84)_ = 14.35, *P *< 0.0001) of *S. calcitrans*. We obtained the largest wing centroid size (Fig. 2a.i), wing length (Fig. 2a.ii), wing width (Fig. 2a.iii), wing area (Fig. 2a.iv), adult weight (Fig. 2a.v) and wing loading (Fig. 2a.vi) in flies reared from a group of five larvae. The biplot from the principal component analysis separated flies reared in a group of five from those reared in a group of 25; flies reared in a group of 15 occupied an intermediate position (Fig. 2a). The two first dimensions accounted for 85.6% of the total wing size variation. Dimension 1 explained 60.6% of the total variation, with wing length as the major contributor. Dimension 2 accounted for 25% of the total variation, with wing loading as the major contributor. Except for an absence of correlation between adult weight and centroid size (*r* = 0.18, *P* = 0.094), and adult weight and wing area (*r* = 0.02, *P* = 0.83), all other parameters were significantly correlated either negatively or positively (Table 2). For instance, wing loading was positively correlated with adult weight (*r* = 0.50, *P *< 0.0001) and negatively correlated with wing width (*r* = − 0.43, *P *< 0.0001), wing length (*r* = − 0.46, *P *< 0.0001), centroid size (*r* = − 0.50, *P *< 0.0001), and wing area (*r* = − 0.60, *P *< 0.0001).

**Table 2:** Correlation matrix between body weight and wing size parameters of *S. calcitrans* reared at different densities. Values above the diagonal represent *P*-values from Pearson correlation tests; values below the diagonal represent correlation coefficient. *P*-value in bold is not significant

	Wing area	Adult weight	Wing width	Wing length	Wing loading	Centroid size
Wing area	–	0.83	< 0.001	< 0.001	< 0.001	< 0.001
Adult weight	0.02	–	0.016	0.024	< 0.001	**0.094**
Wing width	0.54	0.25	–	< 0.001	< 0.001	< 0.001
Wing length	0.57	0.24	0.97	–	< 0.001	< 0.001
Wing loading	− 0.6	0.49	− 0.43	− 0.46	–	< 0.001
Centroid size	0.55	0.18	0.89	0.93	− 0.49	–

#### Wing shape parameters

**Fig. 3:**
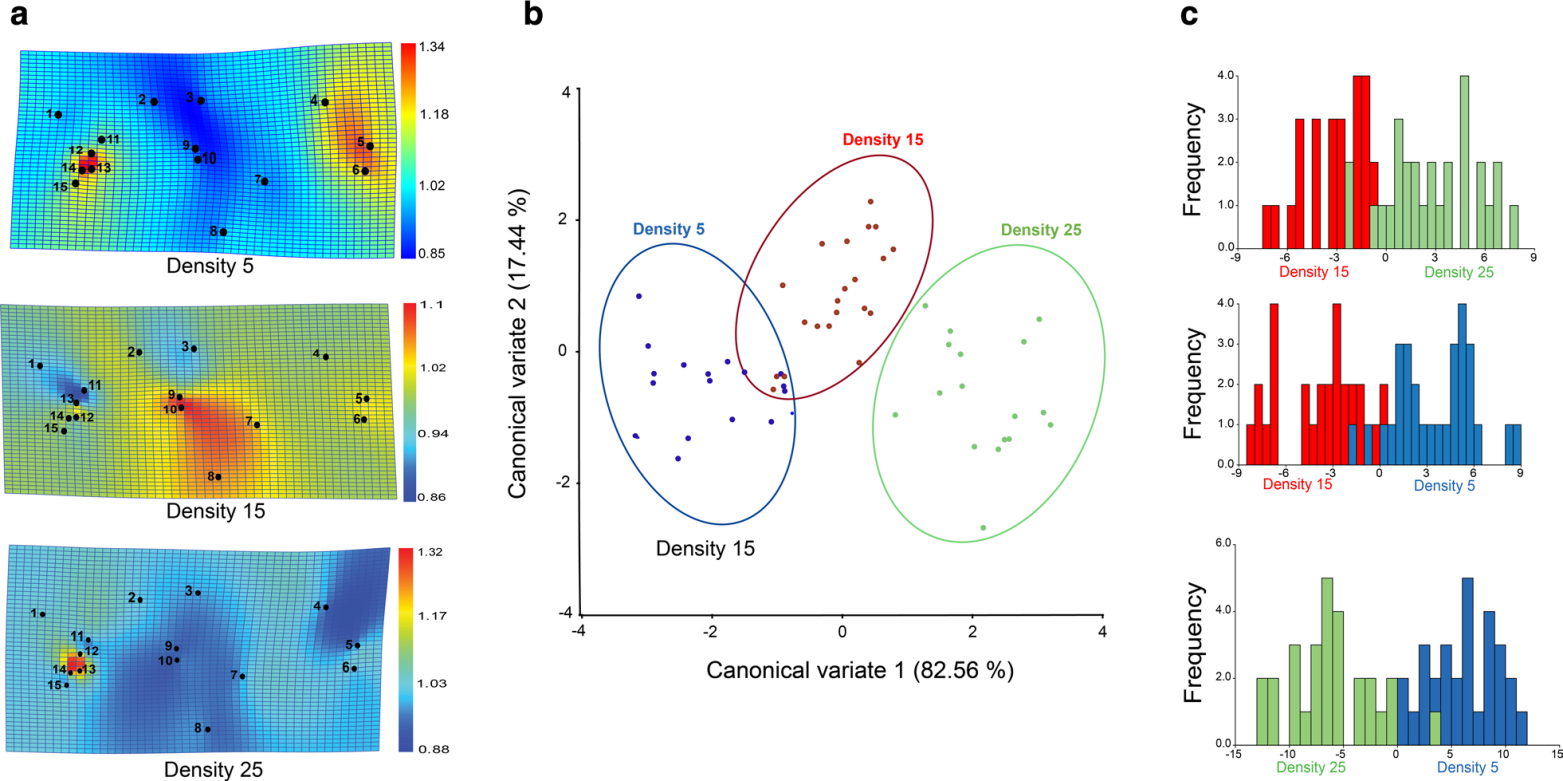
Wing shape of *S. calcitrans* is significantly affected by larval density. **a** Thin plate spline deformation grids modelling the difference of *S. calcitrans* wing shape across the different larval densities. The number displayed on each grid represents the landmark positions. Yellow to orange-red colours indicate landmark expansions, light to dark-blue indicates landmark contraction. **b** Scatter plot showing the difference in wing shapes of *S. calcitrans* individuals reared at different larval densities along the first two canonical variate axes [CV1 (82.56%) and CV2 (17.44%)] with 90% confidence ellipses. **c** Discrimination histograms comparing the *S. calcitrans* wing shape between pairs of larval densities

The wing shape of *S. calcitrans* significantly differed between the larval densities (MANOVA, *F*_(52,1872)_ = 2.26; Pillai’s trace = 0.99; *P* = 0.0059). The thin plate spline (Fig. 3a) showed the variation in expansions and contractions in wing vein intersections (landmarks) of flies emerged from densities of 5, 15 and 25. For instance, in flies from densities of 5 and 15, the landmarks 4, 5 and 6 underwent expansion movement, while in the flies from a density of 25, the same landmarks contracted. Additionally, in flies from densities of 5 and 25, expansions in the landmarks 12, 13 and 14 were more pronounced (red coloured) compared to those of flies from a density of 15 (yellow coloured). The landmarks 7, 8, 9 and 10 expanded in wings emerged from a density of 15, while in fly wings from densities of 5 and 25, these landmarks contracted.

Canonical variate analysis discriminated flies emerged from each density based on wing shape (Fig. 3b). The two first dimensions accounted for 100% of the total shape variation (CV1 = 82.56% and CV2 = 17.44%), and clustered wing shapes in three distinct groups based on the three larval densities. Pairwise comparisons using discriminant analysis with Mahalanobis distances revealed a highly significant difference in *S. calcitrans* wing shapes (Fig. 3c; Table 3; permutation test, 10000 replicates, *P *< 0.0001). When Procrustes distances were used, we found that wing shape of flies reared from densities of 15 and 25 were similar (*P* = 0.16). Regression of Procrustes coordinates on centroid size between densities was significant (permutation test with 10000 rounds, *P* = 0.008), with allometry explaining 2.97% of the total shape variation.

**Table 3:** Difference in the shape of right wings from *S. calcitrans* reared at a density 5, 15 and 25. *P*-values (above the diagonal); distances between populations (below the diagonal). *P *< 0.05 denotes a significant difference

	Mahalanobis distances			Procrustes distances		
	Density 5	Density 15	Density 25	Density 5	Density 15	Density 25
Density 5	–	< 0.001	< 0.001	–	0.0471	< 0.001
Density 15	2.01	–	< 0.001	0.0086	–	0.16
Density 25	3.38	2.23	–	0.0134	0.0083	–

### Effect of developmental substrate on the wing geometry of *S. calcitrans*

#### Wing size parameters

**Fig. 4:**
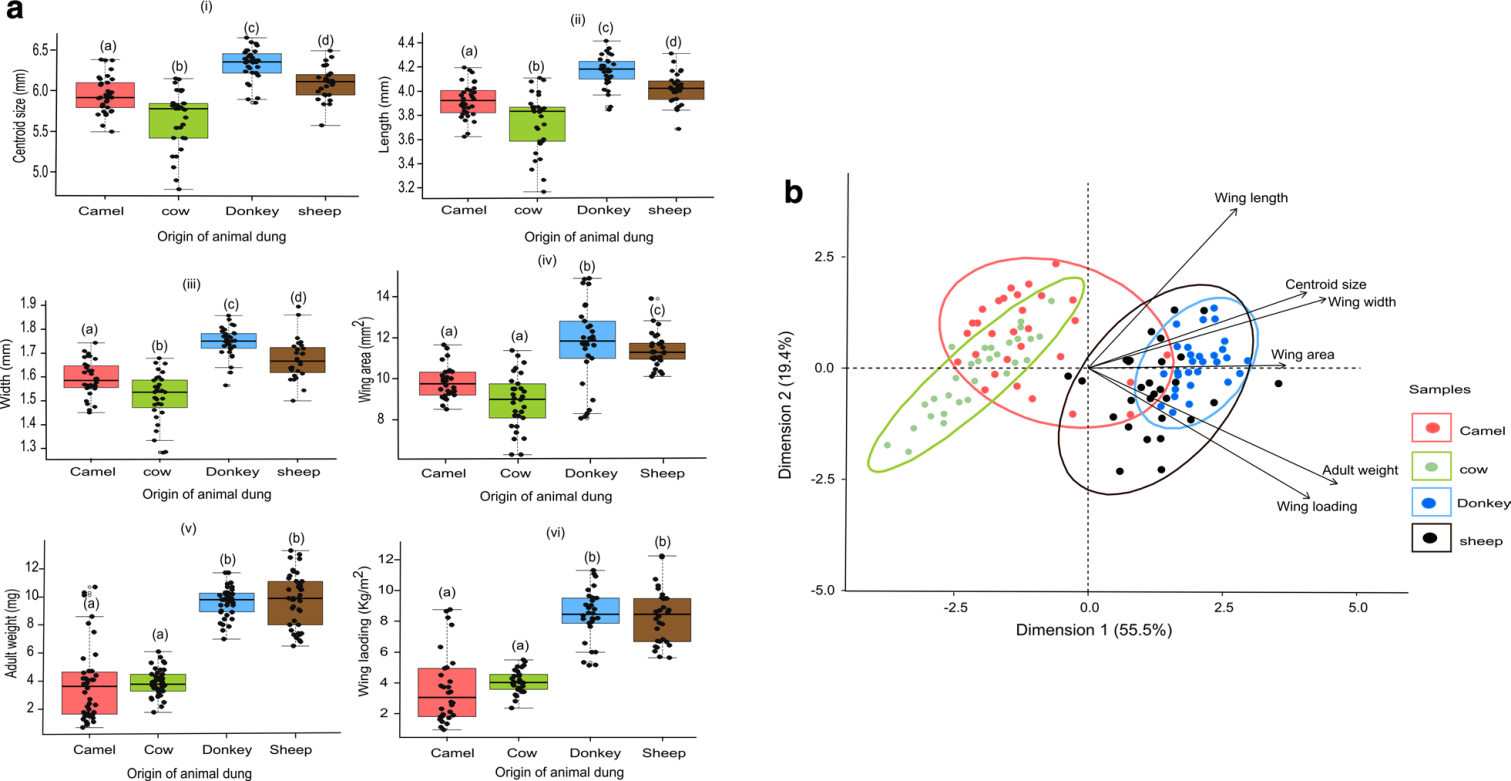
Wing size and adult weight of *S. calcitrans* significantly varies across different developmental substrates. **a** Boxplots depicting variation in wing centroid size (i), wing length (ii), wing width (iii), wing area (iv), adult weight (v), and wing loading (vi) across the different dung substrates. The limit of each boxplot whiskers represents the minimum and maximum of all the data. Boxplots with different letters depict significant differences as grouped by ANOVA tests followed by SNK *post-hoc* tests (*P *< 0.05*, n* = 30). **b** Principal components biplot showing the similarities (or dissimilarities) existing among flies reared from different dung types

Wing centroid size (Fig. 4a.i; *F*_(3,122)_ = 39.24, *P *< 0.0001), wing length (Fig. 4a.ii; *F*_(3,122)_ = 34.9, *P *< 0.0001), wing width (Fig. 4a.iii; *F*_(3,122)_ = 46.98, *P *< 0.0001), wing area (Fig. 4a.iv; *F*_(3,122)_ = 31.02, *P*<0.0001), adult weight (Fig. 4a.v; *F*_(3,122)_ = 140.4, *P *< 0.0001) and the wing loading (Fig. 4a.vi; *F*_(3,122)_ = 67.79, *P *< 0.0001) of *S. calcitrans* individuals reared from various animal dung differed significantly. All these parameters were highest in flies reared on donkey and sheep dung. The principal components analysis differentiated flies emerged from the different herbivore dung (Fig. 4b). The first dimension accounted for 55.5% of the variation in wing shape and were highly correlated with adult weight. The second dimension explained 19.4% of the total variation and was highly associated with wing length. Other than a lack of correlation between wing loading and wing length (*r* = 0.16, *P* = 0.08), all wing size parameters were positively correlated (Table 4).

**Table 4:** Correlation matrix between body weight and wing size parameters of *S. calcitrans* reared from different developmental substrates. Values above the diagonal represent *P*-values from Pearson correlation tests; values below the diagonal represent correlation coefficient. *P*-value in bold is not significant

	Centroid size	Wing length	Wing width	Wing area	Wing loading	Wing mass
Centroid size	1	< 0.001	< 0.001	< 0.001	< 0.001	< 0.001
Wing length	0.43	1	< 0.001	0.01	**0.08**	0.035
Wing width	0.66	0.52	1	< 0.001	< 0.001	< 0.001
Wing area	0.46	0.23	0.49	1	< 0.001	< 0.001
Wing loading	0.39	0.16	0.47	0.3	1	< 0.001
Wing mass	0.46	0.19	0.54	0.57	0.95	1

#### Wing shape parameter

**Fig. 5:**
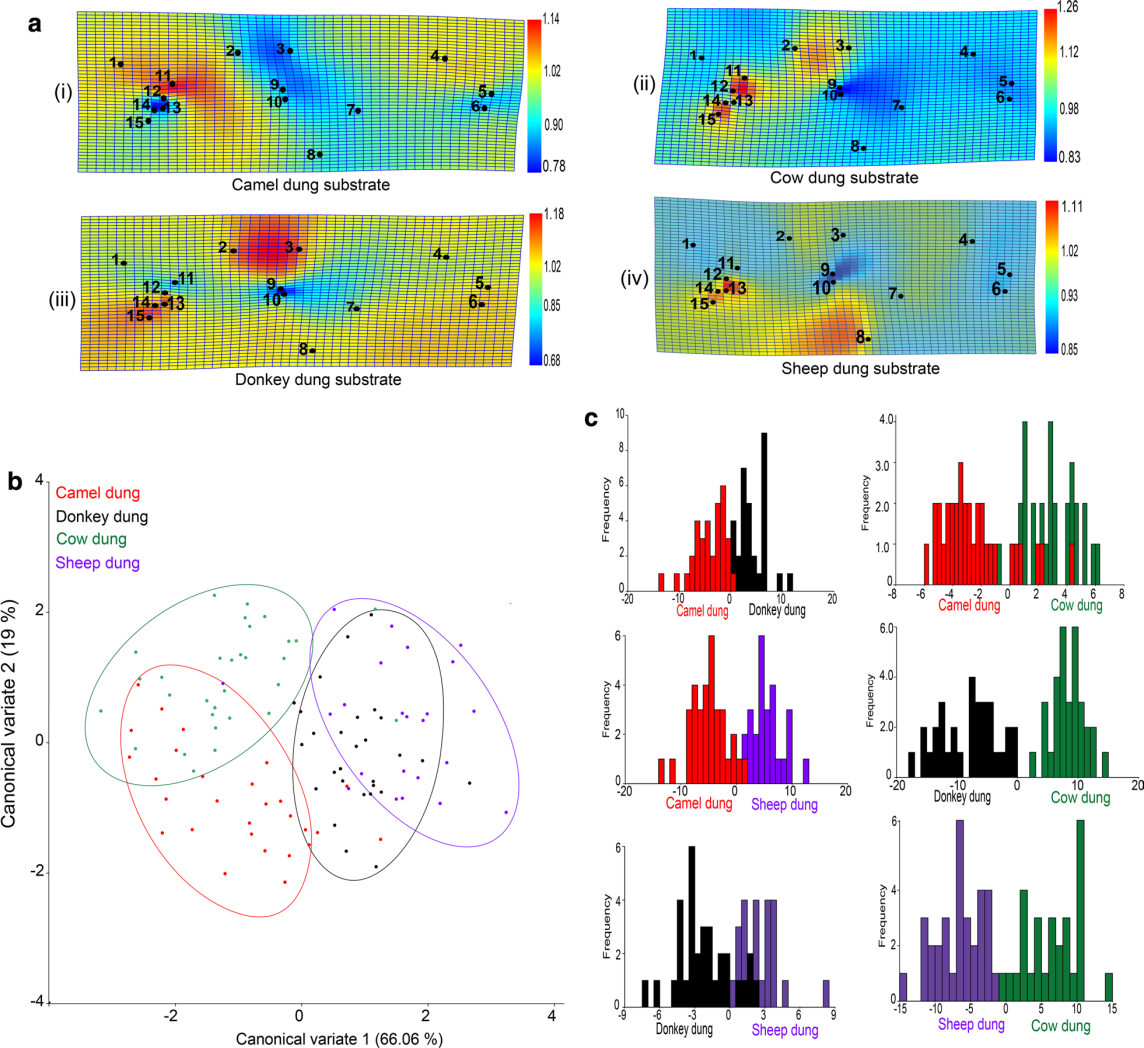
Wing shape in *S. calcitrans* is significantly affected by developmental substrate. **a** Thin plate spline (TPS) deformation grids modelling the difference of *S. calcitrans* wing shape across the different larval densities. The number displayed on each grid represents the landmark positions. Yellow to orange-red colours indicate landmark expansions, light to dark-blue indicates landmark contraction. **b** Scatter plot showing the difference in wing shapes of *S. calcitrans* individuals reared from different developmental substrates along the first two canonical variate axes [CV1 (66.06%) and CV2 (19)] with 90% confidence ellipses. **c** Discrimination histograms comparing *S. calcitrans* wing shape between developmental substrate pairs

Developmental substrate significantly affected the wing shape of *S. calcitrans* (MANOVA, *F*_(78,3172)_ = 4.07, Pillai’s trace = 1.17, *P *< 0.0001). This is clearly illustrated in the thin plate spline deformation grid (Fig. 5a). For instance, in flies emerged from camel dung (Fig. 5a.i), landmarks 12, 13, 14 and 15 underwent expansion movement, whereas in flies from cow (Fig. 5a.ii), donkey (Fig. 5a.iii) and sheep (Fig. 5a.iv) dung, the same landmarks contracted. Canonical variate analysis (Fig. 5b) and discriminant analysis (Fig. 5c) separated *S. calcitrans* wing shapes according to the dung in which flies developed. The first two dimensions of the canonical variate analysis explained 85.06% of the total *S. calcitrans* wing shape variation (Fig. 5b; CV1 = 66.06% and CV2 = 16%). All pairwise permutation tests performed with Mahalanobis distances revealed that the shape of *S. calcitrans* wings diverged significantly when reared from the different animal dung (Table 5; 10,000 rounds, *P *< 0.0001). With Procrustes distance estimators, we obtained a non-significant difference in wing shapes only in flies emerged from camel and cow dung (*P* = 0.2). In the allometry test, the centroid size had a significant effect on wing shape (10,000 rounds of permutation tests, *P* = 0.0015), with a variance prediction of 2.45%.

**Table 5:** Difference in the shape of right wings from *S. calcitrans* reared from various animal dung. *P*-values (above the diagonal); distances between populations (below the diagonal). *P *<  0.05 denotes significant difference

	Mahalanobis distances				Procrustes distances			
	Camel dung	Cow dung	Donkey dung	Sheep dung	Camel dung	Cow dung	Donkey dung	Sheep dung
Camel dung	–	< 0.0001	< 0.0001	< 0.0001	–	0.20	0.0002	< 0.0001
Cow dung	1.7975	–	< 0.0001	< 0.0001	0.0067	–	< 0.0001	< 0.0001
Donkey dung	2.2631	2.3678	–	< 0.0001	0.0109	0.0125	–	0.0476
Sheep dung	3.0651	3.1064	1.9544	–	0.0155	0.0153	0.0081	–

## Discussion

Our study showed that the size and shape of *S. calcitrans* wings exhibit a plastic response to larval density and the quality of the larval substrate. The study also demonstrated the power of the wing landmark-based geometric approach for studying phenotypic plasticity.

We showed that *S. calcitrans* wing size parameters (centroid size, length, width, area, and loading) are affected by larval density (5, 15 and 25) and substrate type (camel, cow, donkey and sheep dung). This indicates the effect of larval developmental conditions on adult wing size. Our study is consistent with previous work on *Ae. aegypti* that revealed the influence of larval density and substrate quality on wing size [47, 48]. The wing size variation obtained in our study may be due to variability in nutrients. Baleba et al. [39] previously determined that donkey and sheep dung, from which emerging flies had the largest wing size, had higher concentrations of specific micronutrients (nitrogen, phosphorous, potassium and zinc) in comparison with camel and cow dung. Furthermore, competition for limited nutrients by *S. calcitrans* larvae may also affect wing size. Dutra et al. [49] found that under high larval density, *Wolbachia*-uninfected *Ae. aegypti* presented reduced wing size (centroid size) and a lower body glucose concentration.

Wing sizes are closely related to the flight capacity in insects. Individuals with longer wings are better at flying compared to those with shorter wings [14]. Long wings favour wider variation in speed and long flight duration [50]. We obtained longer wings in *S. calcitrans* reared in a group of five or when reared from donkey and sheep dung. Long wings allow insects to fly at a great speed for a long period of time and cover a large area [51, 52]. For instance, released *Ae. aegypti* with larger wings are more successful in host-seeking and oviposition site location [53–55]. We predict that *S. calcitrans* developed under lower density conditions are more likely to have good flight capacity. Also, developmental substrates such as donkey and sheep dung may increase the efficiency of *S. calcitrans* flight. This result supports Baleba et al. [39], who found that dung types preferred by female *S. calcitrans* for oviposition were best for offspring growth and development. In this case, preferred substrates lead to potential adult fitness benefits associated with wing size. Low larval density leads to larger adults with long wings due to higher resource availability for growth and nutrient storage in the larval stage. Similarly, development of larvae in preferred dung types would lead to larger adults with larger wings. Such changes may influence dispersal, mating, and vector competency of *S. calcitrans*, rendering this fly more capable of spreading pathogens.

Our study also showed that larval density and substrate type affected *S. calcitrans* wing shape. The thin-plate spline analyses showed that most of the shape changes (landmark movements) occurred on the radial (landmarks 11, 12, 13, 14 and 15) and medial (2, 3, 7, 8, 9 and 10) portions of the *S. calcitrans* wing. Oguz et al. [56] observed the same variation in the radial portion of the wing of *Phlebotomus tobbi* Adler & Theodore. Pieterse et al. [59] found variation in wing landmarks located at the costal, sub-costal and radial veins of *Bactrocera dorsalis* (Hendel) and *Ceratitis capitata* (Wiedemann) reared from nectarine, plum, pear and apple. According to Wootton et al. [57] and Shimmi et al. [58], the radial and the medial portions of insect wings play a critical function in the aerodynamics of insect flight. Wootton [60] suggested such changes may influence the wing strength, beat pattern and ultimately the dispersal potential of a fly. Therefore, the wing shape deformation observed here may affect the flight performance of *S. calcitrans*, their ability to find a host for a blood meal, and consequently, vectorial capacity. Several studies with no emphasis on wing morphology have already demonstrated the indirect effect of larval density and food quality on vector competence. For instance, in *Ae. albopictus*, a greater dissemination rate of Sindbis virus by the adult is the consequence of high levels of competition experienced by the larvae [61]. In *Anopheles stephensi* Liston, larvae developed in a nutritious substrate are more likely to transmit the human malaria parasite, *Plasmodium falciparum*, than those developed in a substrate with a poor nutritional value [62]. The discriminant factors on which the differentiation between flies reared from different density or dung type was based were not free of some allometric effects. In other words, wing size contributed significantly to wing shape variation. However, in the case of both larval density and developmental substrate, less than 3% of variation in wing shape was attributed to size. Such low residual variation indicates that changes in the relative position of landmarks as wing size increases are minimal [63].

## Conclusions

This study highlights the effect of larval density and developmental substrate of wing size and wing shape of *S. calcitrans* using the landmark-based geometric morphometric method. The method satisfactorily discriminated *S. calcitrans* emerged from different larval densities and substrates based on the size and the shape of their wings. While there was a significant effect of size variation on variation in shape, but this accounted for less than 3% of variation. Future studies on flight performance of *S. calcitrans* as well as their vectorial capacity in pathogen transmission when reared under different larval conditions are required. However, our results demonstrate a role for larval density and developmental substrate to influence wing size and to some extent wing shape, which might have a significant effect on flight and dispersal of adult *S. calcitrans*.

## Abbreviations

ANOVA: Analysis of variance
MANOVA: Multivariate analysis of variance
L: Light
D: Dark
